# Distribution of serotypes and antibiotic resistance profiles of *Streptococcus pneumoniae* in hospitalized adult patients: aretrospective multicenter surveillance in China (2018–2019)

**DOI:** 10.1186/s12879-025-11377-5

**Published:** 2025-08-05

**Authors:** Chunjiang Zhao, Feifei Zhang, Zhanwei Wang, Shuo Yang, Hongbin Chen, Hui Wang

**Affiliations:** Department of Clinical Microbiology, Peking University People’s Hospital, Beijing, China

**Keywords:** *Streptococcus pneumoniae*, Serotype, Antimicrobial resistance, Pneumococcal conjunctive vaccine, Adult, China, PCV20

## Abstract

**Background:**

*Streptococcus pneumoniae* (*S. pneumoniae*) remains a major cause of community acquired pneumonia (CAP), particularly among older adults. In China, pneumococcal infections pose a substantial disease burden, with rising antibiotic resistance. Although vaccines have effectively reduced disease worldwide, data on adult serotype distribution and resistance in China are scarce. This study investigates serotype patterns and antimicrobial susceptibility of *S. pneumoniae* among Chinese adults.

**Methods:**

We collected a total of 474 *S. pneumoniae* isolates from adult patients diagnosed with pneumococcal infections in 14 cities across five geographic regions of China between 2018 and 2019. Clinical specimens included sputum, blood, bronchoalveolar lavage fluid (BALF), cerebrospinal fluid (CSF), pharyngeal and nasal swabs, and middle ear fluid. Minimum inhibitory concentrations (MICs) of antimicrobial agents were determined using the agar dilution method. Pneumococcal serotyping was performed using the Pneumotest-Latex kit and type-specific antisera.

**Results:**

Serotype 19F was the most prevalent across all age groups, specimen types, and regions. 20-valent pneumococcal conjugate vaccine (PCV20) is expected to provide the highest serotype coverage (69.4%) among all vaccines evaluated. Multidrug resistance was detected in over 94% of isolates, with high resistance to macrolides, tetracycline, and clindamycin. β-lactam resistance varied by syndrome and region. Non-invasive isolates showed slightly higher resistance than invasive ones. Serotypes covered by higher-valency vaccines were more likely to be antibiotic-resistant, although fluoroquinolone and vancomycin resistance remained low.

**Conclusions:**

Our findings highlight a high burden of antimicrobial resistance and predominance of specific serotypes, particularly 19F, among adult *S. pneumoniae* isolates in China. The broad serotype coverage of PCV20 suggests its potential advantage in future adult immunization strategies. Continued surveillance of serotype distribution and resistance patterns is essential to inform vaccine policy and guide effective antibiotic use.

**Supplementary Information:**

The online version contains supplementary material available at 10.1186/s12879-025-11377-5.

## Introduction

*Streptococcus pneumoniae* (*S. pneumoniae*) is a leading bacterial cause of community acquired pneumonia (CAP), bacteremia, and meningitis worldwide [1]. Although all ages groups are affected, pneumococcal disease significantly affects young children and the elderly, as well as individuals with certain chronic disease and immunocompromising conditions [2]. Globally, pneumococcal-related respiratory infections are associated with a high mortality burden among adults aged over 70 years, with an estimated rate of 44.2 deaths per 100,000 population [3].

In China, *S. pneumoniae* is an important pathogen for morbidity and mortality among children and older adults, and pneumococcal disease cases in China account for 12% of the total pneumococcal cases worldwide [4]. Pneumococcal infections are estimated to cause approximately 89,593 deaths annually among adults older than 70 years in China, with a mortality rate of 82.98 per 100,000 population [5].

Pneumococcal infections are treated with antibiotics; however, treatment may be complicated by the resistance to antibiotic therapies. *S. pneumoniae* isolates from adult patients show high resistance rates to common antibiotics in 2012–2017 (erythromycin, 90.8%; cefuroxime, 58.9%; and ceftriaxone 22.2%). In addition, a high multidrug resistance (MDR) rate was also observed (76.0%) [6].

Immunization with vaccines against *S. pneumoniae* is the most effective public health measure for the prevention of pneumococcal infections. Available pneumococcal vaccines in China include a 23-valent pneumococcal polysaccharide vaccine (PPV23) and a 13-valent pneumococcal conjugate vaccine (PCV13). PPV23 is indicated for use in children aged 2 years or older and in adults [7]. The 7-valent pneumococcal conjugate vaccine (PCV7), targeting serotypes 4, 6B, 9V, 14, 18C, 19F, and 23F, was first licensed in China in 2008 for pediatric immunization. Building on the same conjugation technology, PCV13 expanded coverage to include six additional serotypes (1, 3, 5, 6A, 7F, and 19A) and received regulatory approval in China in 2016, however, exclusively to pediatric populations [8, 9]. More than 100 distinct *S. pneumoniae* serotypes have been identified based on capsular polysaccharide structure, underscoring the complexity of vaccine development [10]. Globally, Multiple countries have introduced PCVs into their national immunization programs for infants and children. Following the implementation of PCVs in these programs, substantial reductions in IPD, pneumonia, and otitis media have been observed in vaccinated children [11–13], as well as in adults through herd immunity [14]. There is a need to directly protect the adults via PCV despite herd protection, therefore, PCV20 and PCV21 were both registered and recommended for adults since 2022, and now are scaling up worldwide [15]. PPV23 vaccination among adults has been shown to effectively alleviate invasive pneumococcal diseases (IPD) burden in adults [16, 17].

This study was designed to investigate the distribution of pneumococcal serotypes and antimicrobial susceptibility of *S. pneumoniae* (2018–2019) to commonly used antimicrobial agents among Chinese adult patients.

## Material and methods

This was a multicenter, retrospective observational study conducted across 14 tertiary hospitals in five regions of China (North, South, East, West, and Central) between January 2018 and December 2019. The study aimed to investigate the serotype distribution and antimicrobial resistance patterns of *Streptococcus pneumoniae* among adult inpatients with confirmed pneumococcal infections.

Consecutive culture-confirmed *S. pneumoniae* isolates were collected from eligible adult patients (≥ 18 years) with clinical diagnoses consistent with pneumococcal infection, including pneumonia, bacteremia, meningitis, and otitis media. Isolates were obtained from sterile and non-sterile sites (e.g., blood, cerebrospinal fluid, sputum, bronchoalveolar lavage fluid), and relevant clinical and demographic data were retrospectively extracted from hospital records.

The study protocol was approved by the Institutional Review Boards of all participating centers, and the use of de-identified retrospective data was granted a waiver of informed consent.

### Bacterial isolates

A total of 474 *S. pneumoniae* isolates were collected from adult patients with pneumococcal infections in 14 cities across 5 regions (North, South, East, West, and Central) of China between 2018 and 2019. The study protocols were approved by Ethics Committee of the Pecking University People’s Hospital and all participants provided written informed consent prior to study commencement. One isolate was collected from each patient. Duplicate isolates and patients colonized by bacteria but without any clinical evidence of infection were excluded. Isolates were obtained from sputum, blood, broncho-alveolar lavage fluid (BALF), cerebrospinal fluid (CSF), pharyngeal swabs, nasal swabs, and middle ear fluid, etc. *S. pneumoniae* isolates from sputum were included if they met the following criteria: less than 10 squamous epithelial cells and more than 25 leukocytes per low power field. Isolates were transported to Peking University People’s Hospital for antibiotic susceptibility testing and serotyping annually. Isolates were identified based on typical colony morphology, Gram staining, optochin sensitivity tests (Oxoid, Hampshire, UK), and Omni serum assays (Statens Serum Institut, Copenhagen, Denmark).

### In vitro antimicrobial susceptibility testing

The agar dilution method was used to determine the minimum inhibitory concentrations (MICs) of the 474 *S. pneumoniae* isolates against 15 antibiotics (penicillin, amoxicillin/clavulanic acid, ceftriaxone, cefuroxime, cefaclor, vancomycin, erythromycin, azithromycin, clarithromycin, tetracycline, levofloxacin, moxifloxacin, trimethoprim/ sulfamethoxazole, chloramphenicol and clindamycin) in accordance with the guidelines established by the Clinical and Laboratory Standards Institute (CLSI). The CLSI 2023 criteria for MICs were applied to classify isolates as susceptible, intermediate, or resistant [18]. The oral penicillin breakpoint was used to classify isolates as penicillin susceptible (MIC ≤ 0.06 μg/ml), penicillin-intermediate (MIC between 0.12 and 1 μg/ml), or penicillin-resistant (MIC ≥ 2 μg/ml). For ceftriaxone, the non-meningitis breakpoint was used to classify isolates as susceptible (MIC ≤ 1 μg/ml) or resistant (MIC ≥ 4 μg/ml)  [17]. *S. pneumoniae* ATCC 49619 was used as the quality control strain and was included in each set of tests to ensure accurate results. Multidrug-resistant (MDR) *S. pneumoniae* isolates were defined as isolates that were resistant to ≥ 3 classes of antimicrobial agents.

### Pneumococcal serotyping

Pneumococcal serotypes/groups were determined for the 474 isolates with Pneumotest-Latex kit (Statens Serum Institut, Copenhagen, Denmark) and type-specific antisera (Statens Serum Institut, Copenhagen, Denmark). The Pneumotest-Latex kit consisted of the 14 latex reagents pools A-I and P-Q. By testing all 14 pools and using the chessboard identification system, it was possible to identify the 23 vaccine serotypes to type/group level. Traditional quellung reaction with type-specific antisera was used for full serotyping of serogroup 19, 6 and 23. The coverages of PCV13, PCV15, PCV20, PCV21 and PPV23 vaccines were estimated by calculating the percentage of isolates that expressed the serotypes included in the vaccines.

### Statistical analysis

Descriptive, non-comparative analysis was used in this study. Frequency distributions that provided the number and percentage of pneumococcal isolates per serotype were calculated for all serotypes identified in this study. Serotypes were grouped by PCV13, PCV15, PCV20, PCV21 and PPV23 coverage. Breakdowns of serotypes by age groups (18–49, 50–59, 60–69, ≥ 70) for above serotype distribution were also presented.

The number and percentage of susceptible, intermediate, and resistant serotypes of *S. pneumoniae* isolates were calculated. Overall frequency distribution of susceptible, intermediate, and resistant serotypes was given for each antibiotic. The data were analyzed using the R software (version 4.4.2).

## Results

### Characteristics of pneumococcal isolates

**Fig. 1:**
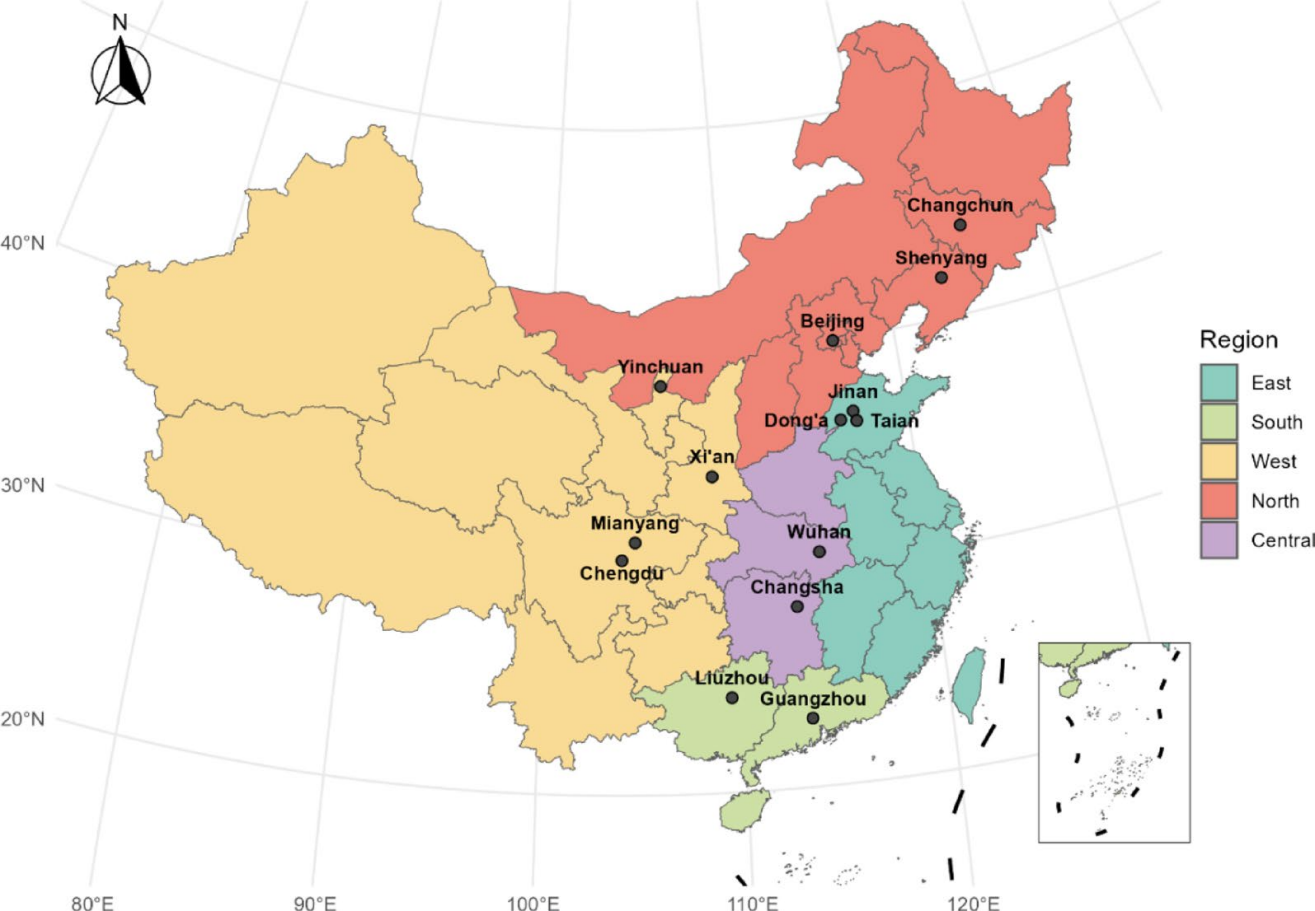
Distribution of 20 general hospitals included in the study

During the study period, a total of 474 *S. pneumoniae* isolates were collected, with 66.9% derived from male patients and 33.1% from females. Patients aged 70 years and older accounted for the largest proportion (35.7%) among all age groups. Most isolates were obtained from non-invasive specimens (89.2%), while 10.8% were from invasive sources such as blood and cerebrospinal fluid. Table S1 presents the basic characteristics of *S. pneumoniae* isolates, categorized into invasive and non-invasive types, with invasive strains detected across all five regions. Geographically, most isolates were collected from northern China (46.2%), followed by the western (23.9%), eastern (15.6%), southern (11.0%), and central (3.4%) regions (Table 1, Fig. 1).

**Table 1:** Characteristics of patients with collected *S. pneumoniae* isolates

Characteristics	No	Percentage (%)
Total	474	100.0
Gender		
Male	317	66.9
Female	157	33.1
Age group (years)		
18–49	72	15.2
50–59	91	19.2
60–69	142	30.0
≥ 70	169	35.7
Specimen source		
Non-invasive	423	89.2
Sputum	334	70.5
Bronchoalveolar lavage	52	11
Nose	14	3
Secretions	5	1.1
Brush biopsy	4	0.8
Sinus	4	0.8
Pus	3	0.6
Urine	3	0.6
Drainage	2	0.4
Ear	1	0.2
Throat swab	1	0.2
Invasive	51	10.8
Blood	41	8.6
Cerebrospinal fluid	7	1.5
Eye (Sterile)	2	0.4
Pleural fluid	1	0.2
Region (city, province)		
East	74	15.6
Dong’a County People’s Hospital (Dong’a, Shandong)	22	4.6
Shandong Provincial Hospital (Jinan, Shandong)	22	4.6
Tai’an Central Hospital (Tai’an, Shandong)	30	6.3
South	52	11.0
Guangzhou Institute of Respiratory Health (Guangzhou, Guangdong)	20	4.2
Liuzhou People’s Hospital (Liuzhou, Guangxi)	30	6.3
Liuzhou Maternal and Child Health Hospital (Liuzhou, Guangxi)	1	0.2
The First Affiliated Hospital of Sun Yat-sen University (Guangzhou, Guangdong)	1	0.2
West	113	23.9
Ningxia Medical University General Hospital (Yinchuan, Ningxia)	28	5.9
The Third Hospital of Mianyang (Mianyang, Sichuan)	42	8.9
West China Hospital of Sichuan University (Chengdu, Sichuan)	22	4.6
Xijing Hospital (Xian, Shannxi)	21	4.4
North	219	46.2
Beijing Tsinghua Chang Gung Memorial Hospital (Beijing)	6	1.3
Beijing Chaoyang Hospital (Beijing)	79	16.7
China-Japan Friendship Hospital (Beijing)	40	8.4
China Medical University Shengjing Hospital (Shenyang, Liaoning)	19	4.0
Jilin University Sino-Japanese Friendship Hospital (Changchun, Jilin)	3	0.6
Peking University People’s Hospital (Beijing)	72	15.2
Central	16	3.4
Wuhan Fourth Hospital (Wuhan, Hubei)	13	2.7
Xiangya Hospital Central South University (Changsha, Hunan)	1	0.2
Hunan Provincial People’s Hospital (Changsha, Hunan)	2	0.4

### Serotype distribution

**Fig. 2:**
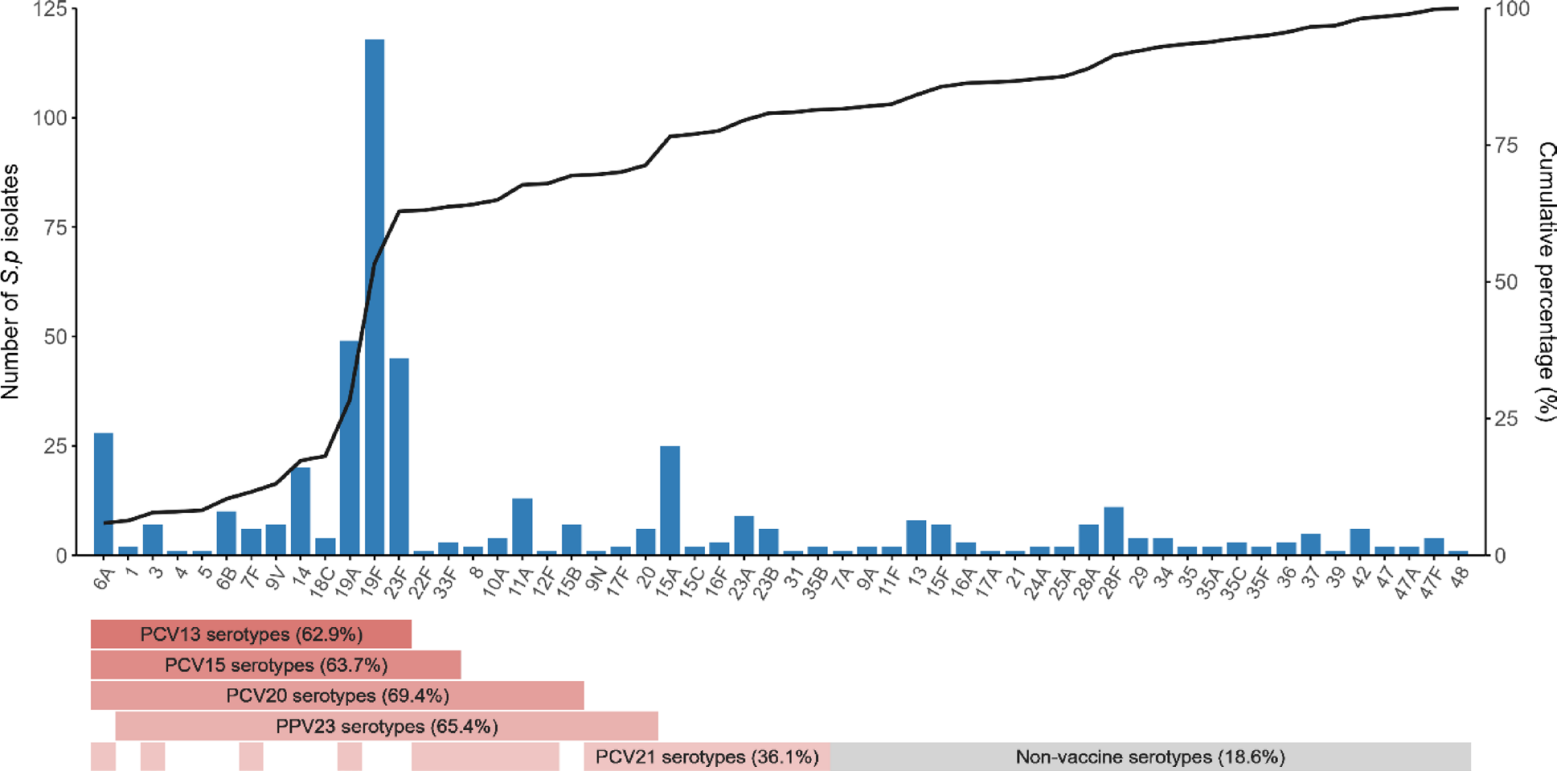
Serotype distribution of *S.p* isolates

**Fig. 3:**
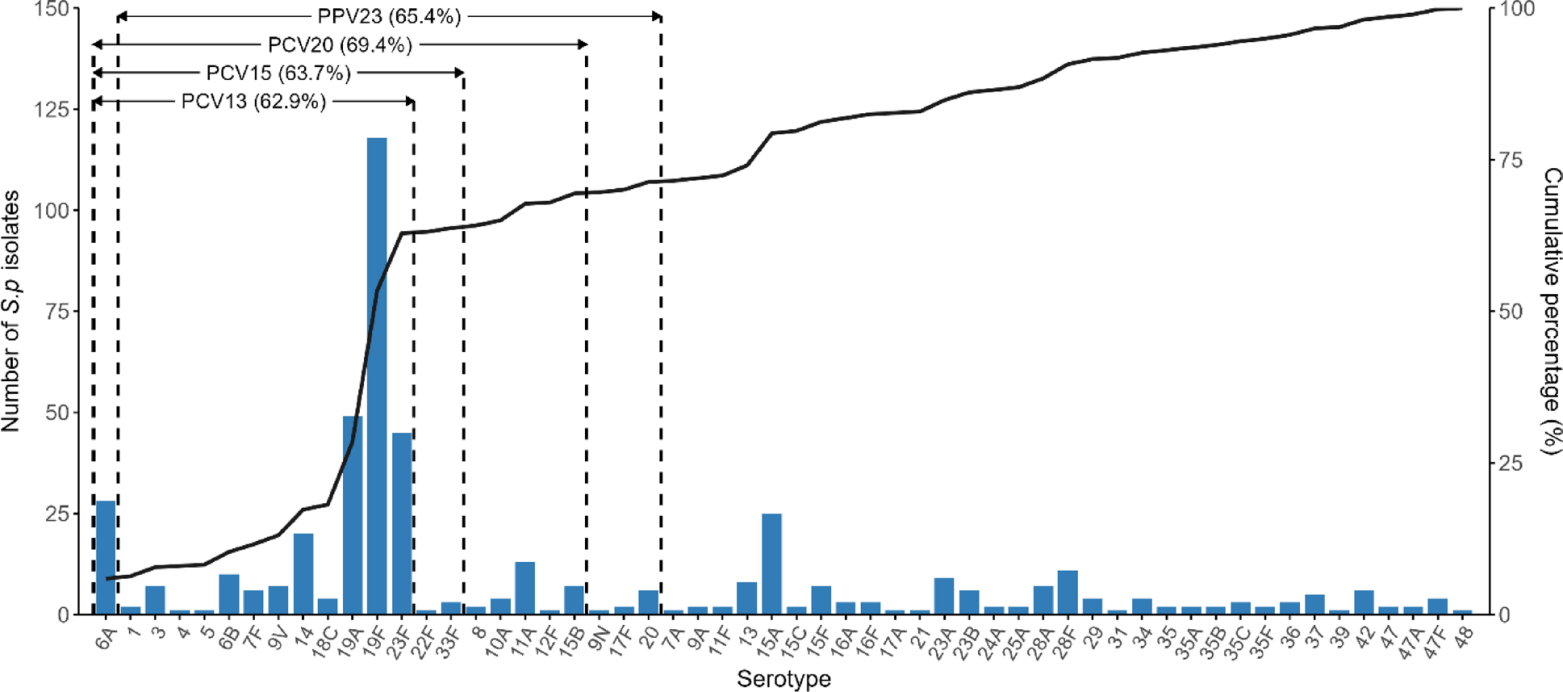
Serotype distribution of *S.p* isolates

The serotype distribution of the isolates is presented in Table 2. Overall, serotype 19F was the most prevalent (24.9%), followed by 19A (10.3%), 23F (9.5%), 6A (5.9%), and 15A (5.3%) (Figs. 2 and 3). Across all age groups, 19F remained the predominant serotype, ranging from 16.9% (50–59 years) to 30.2% (≥ 70 years). Similarly, 19F was the most prevalent serotype across all geographic regions, ranging from 22.4% (northern) to 31.2% (central). In the eastern and western regions, 19A was the second most common serotype, accounting for 16.2% and 13.3% of isolates, respectively, whereas in the southern, northern, and central regions, 23F ranked second (13.5%, 9.6%, and 12.5%, respectively). Serotype 19F maintained dominance in both invasive and non-invasive specimens, with respective prevalence rates of 27.5% and 24.6%. Notably, among invasive isolates, serotype 6A (11.8%) emerged as the secondary variant, closely trailing 19F. The proportion of serotype 33F, a PCV20-nonPCV13 serotype, was significantly higher in the invasive group (3.9%) compared to the non-invasive group (0.2%) (*P* < 0.05) (Table S2). A parallel pattern was observed in older adults (60–69 years), where 19F retained prominence at 16.9%, while 6A constituted the second most prevalent serotype (11.3%).

**Table 2:** Serotype distribution of *S.pneumoniae* [n (%)]

Serotype	Total (n = 474)	Age group					Region				Specimen source	
		18–49 (n = 72)	50–59 (n = 91)	60–69 (n = 142)	≥ 70 (n = 169)	East (n = 74)	South (n = 52)	West (n = 113)	North (n = 219)	Central (n = 16)	Invasive (n = 51)	Non-invasive (n = 423)
Vaccine												
1	2 (0.4)	0 (0.0)	0 (0.0)	1 (0.7)	1 (0.6)	0 (0.0)	1 (1.9)	0 (0.0)	1 (0.5)	0 (0.0)	1 (2.0)	1 (0.2)
3	7 (1.5)	1 (1.4)	4 (4.4)	0 (0.0)	2 (1.2)	1 (1.4)	0 (0.0)	1 (0.9)	4 (1.8)	1 (6.2)	1 (2.0)	6 (1.4)
4	1 (0.2)	1 (1.4)	0 (0.0)	0 (0.0)	0 (0.0)	0 (0.0)	1 (1.9)	0 (0.0)	0 (0.0)	0 (0.0)	1 (2.0)	0 (0.0)
5	1 (0.2)	0 (0.0)	0 (0.0)	0 (0.0)	1 (0.6)	1 (1.4)	0 (0.0)	0 (0.0)	0 (0.0)	0 (0.0)	0 (0.0)	1 (0.2)
6A	28 (5.9)	2 (2.8)	7 (7.7)	16 (11.3)	3 (1.8)	5 (6.8)	2 (3.8)	8 (7.1)	13 (5.9)	0 (0.0)	6 (11.8)	22 (5.2)
6B	10 (2.1)	0 (0.0)	1 (1.1)	4 (2.8)	5 (3.0)	4 (5.4)	2 (3.8)	2 (1.8)	2 (0.9)	0 (0.0)	1 (2.0)	9 (2.1)
7F	6 (1.3)	1 (1.4)	0 (0.0)	3 (2.1)	2 (1.2)	1 (1.4)	0 (0.0)	3 (2.7)	2 (0.9)	0 (0.0)	1 (2.0)	5 (1.2)
8	2 (0.4)	0 (0.0)	0 (0.0)	1 (0.7)	1 (0.6)	0 (0.0)	0 (0.0)	0 (0.0)	2 (0.9)	0 (0.0)	0 (0.0)	2 (0.5)
9N	1 (0.2)	0 (0.0)	0 (0.0)	1 (0.7)	0 (0.0)	0 (0.0)	0 (0.0)	0 (0.0)	1 (0.5)	0 (0.0)	0 (0.0)	1 (0.2)
9V	7 (1.5)	0 (0.0)	3 (3.3)	1 (0.7)	3 (1.8)	2 (2.7)	0 (0.0)	0 (0.0)	4 (1.8)	1 (6.2)	1 (2.0)	6 (1.4)
10A	4 (0.8)	1 (1.4)	0 (0.0)	1 (0.7)	2 (1.2)	0 (0.0)	0 (0.0)	1 (0.9)	3 (1.4)	0 (0.0)	0 (0.0)	4 (0.9)
11A	13 (2.7)	4 (5.6)	4 (4.4)	4 (2.8)	1 (0.6)	4 (5.4)	2 (3.8)	2 (1.8)	4 (1.8)	1 (6.2)	1 (2.0)	12 (2.8)
12F	1 (0.2)	1 (1.4)	0 (0.0)	0 (0.0)	0 (0.0)	0 (0.0)	1 (1.9)	0 (0.0)	0 (0.0)	0 (0.0)	0 (0.0)	1 (0.2)
14	20 (4.2)	4 (5.6)	2 (2.2)	7 (4.9)	7 (4.1)	3 (4.1)	2 (3.8)	4 (3.5)	11 (5.0)	0 (0.0)	3 (5.9)	17 (4.0)
15A	25 (5.3)	0 (0.0)	7 (7.7)	10 (7.0)	8 (4.7)	3 (4.1)	3 (5.8)	11 (9.7)	7 (3.2)	1 (6.2)	0 (0.0)	25 (5.9)
15B	7 (1.5)	2 (2.8)	1 (1.1)	2 (1.4)	2 (1.2)	1 (1.4)	0 (0.0)	1 (0.9)	5 (2.3)	0 (0.0)	1 (2.0)	6 (1.4)
15C	2 (0.4)	0 (0.0)	0 (0.0)	1 (0.7)	1 (0.6)	0 (0.0)	0 (0.0)	1 (0.9)	1 (0.5)	0 (0.0)	0 (0.0)	2 (0.5)
16F	3 (0.6)	0 (0.0)	0 (0.0)	0 (0.0)	3 (1.8)	1 (1.4)	0 (0.0)	1 (0.9)	1 (0.5)	0 (0.0)	0 (0.0)	3 (0.7)
17F	2 (0.4)	0 (0.0)	0 (0.0)	1 (0.7)	1 (0.6)	0 (0.0)	0 (0.0)	0 (0.0)	2 (0.9)	0 (0.0)	1 (2.0)	1 (0.2)
18C	4 (0.8)	0 (0.0)	0 (0.0)	1 (0.7)	3 (1.8)	2 (2.7)	0 (0.0)	0 (0.0)	2 (0.9)	0 (0.0)	0 (0.0)	4 (0.9)
19A	49 (10.3)	7 (9.7)	9 (9.9)	14 (9.9)	19 (11.2)	12 (16.2)	1 (1.9)	15 (13.3)	20 (9.1)	1 (6.2)	6 (11.8)	43 (10.2)
19F	118 (24.9)	16 (22.2)	27 (29.7)	24 (16.9)	51 (30.2)	20 (27.0)	15 (28.8)	29 (25.7)	49 (22.4)	5 (31.2)	14 (27.5)	104 (24.6)
20	6 (1.3)	0 (0.0)	2 (2.2)	0 (0.0)	4 (2.4)	0 (0.0)	0 (0.0)	1 (0.9)	4 (1.8)	1 (6.2)	0 (0.0)	6 (1.4)
22F	1 (0.2)	0 (0.0)	0 (0.0)	0 (0.0)	1 (0.6)	0 (0.0)	0 (0.0)	1 (0.9)	0 (0.0)	0 (0.0)	0 (0.0)	1 (0.2)
23A	9 (1.9)	3 (4.2)	0 (0.0)	1 (0.7)	5 (3.0)	1 (1.4)	3 (5.8)	1 (0.9)	4 (1.8)	0 (0.0)	1 (2.0)	8 (1.9)
23B	6 (1.3)	1 (1.4)	1 (1.1)	3 (2.1)	1 (0.6)	0 (0.0)	1 (1.9)	1 (0.9)	4 (1.8)	0 (0.0)	1 (2.0)	5 (1.2)
23F	45 (9.5)	11 (15.3)	8 (8.8)	14 (9.9)	12 (7.1)	5 (6.8)	7 (13.5)	10 (8.8)	21 (9.6)	2 (12.5)	2 (3.9)	43 (10.2)
31	1 (0.2)	0 (0.0)	0 (0.0)	0 (0.0)	1 (0.6)	0 (0.0)	0 (0.0)	0 (0.0)	1 (0.5)	0 (0.0)	0 (0.0)	1 (0.2)
33F	3 (0.6)	0 (0.0)	0 (0.0)	3 (2.1)	0 (0.0)	0 (0.0)	0 (0.0)	0 (0.0)	3 (1.4)	0 (0.0)	2 (3.9)	1 (0.2)
35B	2 (0.4)	0 (0.0)	0 (0.0)	1 (0.7)	1 (0.6)	0 (0.0)	0 (0.0)	1 (0.9)	1 (0.5)	0 (0.0)	0 (0.0)	2 (0.5)
Non-vaccine												
7A	1 (0.2)	0 (0.0)	0 (0.0)	0 (0.0)	1 (0.6)	1 (1.4)	0 (0.0)	0 (0.0)	0 (0.0)	0 (0.0)	0 (0.0)	1 (0.2)
9A	2 (0.4)	0 (0.0)	1 (1.1)	1 (0.7)	0 (0.0)	0 (0.0)	0 (0.0)	1 (0.9)	1 (0.5)	0 (0.0)	0 (0.0)	2 (0.5)
11F	2 (0.4)	1 (1.4)	0 (0.0)	1 (0.7)	0 (0.0)	1 (1.4)	0 (0.0)	0 (0.0)	1 (0.5)	0 (0.0)	0 (0.0)	2 (0.5)
13	8 (1.7)	1 (1.4)	0 (0.0)	2 (1.4)	5 (3.0)	1 (1.4)	0 (0.0)	1 (0.9)	6 (2.7)	0 (0.0)	0 (0.0)	8 (1.9)
15F	7 (1.5)	2 (2.8)	0 (0.0)	3 (2.1)	2 (1.2)	0 (0.0)	0 (0.0)	0 (0.0)	7 (3.2)	0 (0.0)	1 (2.0)	6 (1.4)
16A	3 (0.6)	0 (0.0)	0 (0.0)	1 (0.7)	2 (1.2)	0 (0.0)	0 (0.0)	2 (1.8)	1 (0.5)	0 (0.0)	0 (0.0)	3 (0.7)
17A	1 (0.2)	0 (0.0)	1 (1.1)	0 (0.0)	0 (0.0)	0 (0.0)	0 (0.0)	0 (0.0)	1 (0.5)	0 (0.0)	0 (0.0)	1 (0.2)
21	1 (0.2)	0 (0.0)	0 (0.0)	1 (0.7)	0 (0.0)	0 (0.0)	0 (0.0)	0 (0.0)	0 (0.0)	1 (6.2)	0 (0.0)	1 (0.2)
24A	2 (0.4)	0 (0.0)	1 (1.1)	1 (0.7)	0 (0.0)	0 (0.0)	0 (0.0)	2 (1.8)	0 (0.0)	0 (0.0)	0 (0.0)	2 (0.5)
25A	2 (0.4)	2 (2.8)	0 (0.0)	0 (0.0)	0 (0.0)	0 (0.0)	0 (0.0)	1 (0.9)	1 (0.5)	0 (0.0)	0 (0.0)	2 (0.5)
28A	7 (1.5)	0 (0.0)	1 (1.1)	3 (2.1)	3 (1.8)	0 (0.0)	0 (0.0)	1 (0.9)	5 (2.3)	1 (6.2)	0 (0.0)	7 (1.7)
28F	11 (2.3)	4 (5.6)	2 (2.2)	2 (1.4)	3 (1.8)	1 (1.4)	1 (1.9)	2 (1.8)	7 (3.2)	0 (0.0)	3 (5.9)	8 (1.9)
29	4 (0.8)	1 (1.4)	1 (1.1)	1 (0.7)	1 (0.6)	0 (0.0)	1 (1.9)	1 (0.9)	2 (0.9)	0 (0.0)	0 (0.0)	4 (0.9)
34	4 (0.8)	0 (0.0)	1 (1.1)	1 (0.7)	2 (1.2)	1 (1.4)	2 (3.8)	0 (0.0)	1 (0.5)	0 (0.0)	0 (0.0)	4 (0.9)
35	2 (0.4)	1 (1.4)	0 (0.0)	0 (0.0)	1 (0.6)	1 (1.4)	1 (1.9)	0 (0.0)	0 (0.0)	0 (0.0)	0 (0.0)	2 (0.5)
35A	2 (0.4)	0 (0.0)	0 (0.0)	1 (0.7)	1 (0.6)	0 (0.0)	0 (0.0)	2 (1.8)	0 (0.0)	0 (0.0)	0 (0.0)	2 (0.5)
35C	3 (0.6)	0 (0.0)	2 (2.2)	1 (0.7)	0 (0.0)	0 (0.0)	0 (0.0)	0 (0.0)	2 (0.9)	1 (6.2)	0 (0.0)	3 (0.7)
35F	2 (0.4)	0 (0.0)	1 (1.1)	1 (0.7)	0 (0.0)	0 (0.0)	0 (0.0)	1 (0.9)	1 (0.5)	0 (0.0)	0 (0.0)	2 (0.5)
36	3 (0.6)	1 (1.4)	1 (1.1)	0 (0.0)	1 (0.6)	0 (0.0)	3 (5.8)	0 (0.0)	0 (0.0)	0 (0.0)	0 (0.0)	3 (0.7)
37	5 (1.1)	1 (1.4)	2 (2.2)	1 (0.7)	1 (0.6)	0 (0.0)	0 (0.0)	1 (0.9)	4 (1.8)	0 (0.0)	0 (0.0)	5 (1.2)
39	1 (0.2)	0 (0.0)	0 (0.0)	1 (0.7)	0 (0.0)	0 (0.0)	0 (0.0)	0 (0.0)	1 (0.5)	0 (0.0)	1 (2.0)	0 (0.0)
42	6 (1.3)	1 (1.4)	1 (1.1)	3 (2.1)	1 (0.6)	0 (0.0)	1 (1.9)	2 (1.8)	3 (1.4)	0 (0.0)	0 (0.0)	6 (1.4)
47	2 (0.4)	1 (1.4)	0 (0.0)	0 (0.0)	1 (0.6)	0 (0.0)	1 (1.9)	0 (0.0)	1 (0.5)	0 (0.0)	1 (2.0)	1 (0.2)
47A	2 (0.4)	0 (0.0)	0 (0.0)	2 (1.4)	0 (0.0)	1 (1.4)	0 (0.0)	1 (0.9)	0 (0.0)	0 (0.0)	1 (2.0)	1 (0.2)
47F	4 (0.8)	1 (1.4)	0 (0.0)	1 (0.7)	2 (1.2)	1 (1.4)	1 (1.9)	0 (0.0)	2 (0.9)	0 (0.0)	0 (0.0)	4 (0.9)
48	1 (0.2)	0 (0.0)	0 (0.0)	0 (0.0)	1 (0.6)	0 (0.0)	0 (0.0)	1 (0.9)	0 (0.0)	0 (0.0)	0 (0.0)	1 (0.2)

Vaccine coverage across different groups is presented in Table 3. Among all isolates, PCV20 demonstrated the highest overall coverage (69.4%), followed by PPV23 (65.4%), PCV15 (MSD) (63.7%), and PCV13 (62.9%). PCV21 had the lowest coverage (36.1%), while 18.6% of isolates were not covered by any of the evaluated vaccines. Across all age groups, PCV20 consistently showed the highest coverage, ranging from 67.6% (60–69 years) to 72.5% (50–59 years). Regionally, PCV20 had the highest coverage in all areas except the central region, where PPV23 coverage (75.0%) was the highest. Similarly, PCV20 demonstrated the greatest coverage in both invasive (80.4%) and non-invasive (68.1%) specimens.

**Table 3:** Vaccine coverage across different groups [n (%)]

Vaccine	Total (n = 474)	Age group				Region					Specimen source	
		18–49 (n = 72)	50–59 (n = 91)	60–69 (n = 142)	≥ 70 (n = 169)	East (n = 74)	South (n = 52)	West (n = 113)	North (n = 219)	Central (n = 16)	Invasive (n = 51)	Non-invasive (n = 423)
PCV13	298 (62.9)	43 (59.7)	61 (67.0)	85 (59.9)	109 (64.5)	56 (75.7)	31 (59.6)	72 (63.7)	129 (58.9)	10 (62.5)	37 (72.5)	261 (61.7)
PCV15 (MSD)	302 (63.7)	43 (59.7)	61 (67.0)	88 (62.0)	110 (65.1)	56 (75.7)	31 (59.6)	73 (64.6)	132 (60.3)	10 (62.5)	39 (76.5)	263 (62.2)
PCV15 (Zhifei)	299 (63.1)	44 (61.1)	61 (67.0)	85 (59.9)	109 (64.5)	56 (75.7)	32 (61.5)	72 (63.7)	129 (58.9)	10 (62.5)	37 (72.5)	262 (61.9)
PCV20	329 (69.4)	51 (70.8)	66 (72.5)	96 (67.6)	116 (68.6)	61 (82.4)	34 (65.4)	77 (68.1)	146 (66.7)	11 (68.8)	41 (80.4)	288 (68.1)
PCV21	171 (36.1)	21 (29.2)	34 (37.4)	60 (42.3)	56 (33.1)	28 (37.8)	13 (25.0)	48 (42.5)	77 (35.2)	5 (31.2)	20 (39.2)	151 (35.7)
PPV23	310 (65.4)	49 (68.1)	61 (67.0)	82 (57.7)	118 (69.8)	56 (75.7)	32 (61.5)	70 (61.9)	140 (63.9)	12 (75.0)	36 (70.6)	274 (64.8)
Non-vaccine	88 (18.6)	17 (23.6)	15 (16.5)	28 (19.7)	28 (16.6)	8 (10.8)	11 (21.2)	19 (16.8)	47 (21.5)	3 (18.8)	7 (13.7)	81 (19.1)

### Antibiotic resistance profiles of *S. pneumoniae* across regions

**Fig. 4:**
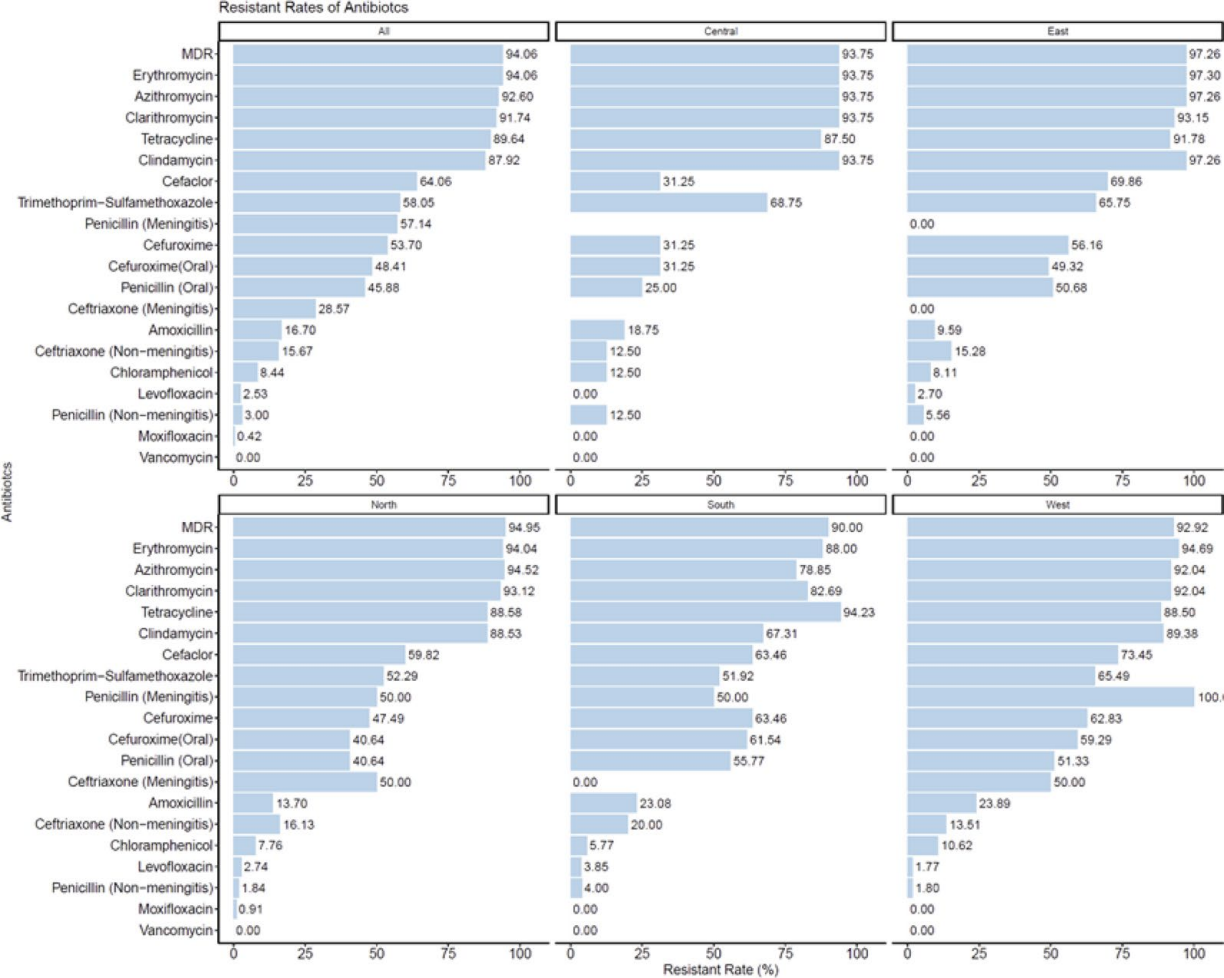
Antibiotic resistance of *S. pneumoniae* by regions

Antibiotic resistance among *S. pneumoniae* isolates from hospitalized adult patients in China was notably high, with multidrug resistance (MDR) detected in 94.06% of cases (Fig. 4). Resistance to macrolides was particularly widespread, with over 90% of isolates exhibiting resistance to erythromycin, azithromycin, and clarithromycin. Similarly, high resistance rates were observed for tetracycline (89.64%) and clindamycin (87.92%). Penicillin resistance was observed in 57.14% of isolates from meningitis cases and 45.81% of those from oral treatment cases. In comparison, resistance to third-generation cephalosporins was lower: ceftriaxone resistance was 28.57% in isolates associated with meningitis and 15.67% in non-meningitis cases. Resistance to trimethoprim-sulfamethoxazole was moderate (58.05%), whereas resistance to amoxicillin (16.70%) and chloramphenicol (8.44%) remained relatively low. Fluoroquinolone resistance was rare, and no resistance to vancomycin was detected.

Regional variations were evident in antibiotic resistance patterns. Macrolide and tetracycline resistance were consistently high across all regions, while β-lactam resistance varied. Notably, the East and West regions exhibited higher resistance rates for several antibiotics, whereas resistance to ceftriaxone (meningitis) was lowest in the East and highest in the West. Chloramphenicol resistance was highest in the Central region. The detailed distribution of antibiotic resistance rates across different regions is shown in Table 4. The comparison of antibiotic resistance profiles between invasive and non-invasive isolates is summarized in Table 5.

**Table 4:** Antibiotic resistance of *S. pneumoniae* by regions

Antibiotic	Central	East	West	South	North	All regions
Penicillin (Meningitis)	–	0.00% (0/1)	100.00% (2/2)	50.00% (1/2)	50.00% (1/2)	57.14% (4/7)
Penicillin (Non-meningitis)	12.50% (2/16)	5.56% (4/72)	1.80% (2/111)	4.00% (2/50)	1.84% (4/217)	3.00% (14/466)
Penicillin (Oral)	25.00% (4/16)	50.68% (37/73)	51.33% (58/113)	55.77% (29/52)	40.64% (89/219)	45.88% (217/473)
Amoxicillin	18.75% (3/16)	9.59% (7/73)	23.89% (27/113)	23.08% (12/52)	13.70% (30/219)	16.70% (79/473)
Cefaclor	31.25% (5/16)	69.86% (51/73)	73.45% (83/113)	63.46% (33/52)	59.82% (131/219)	64.06% (303/473)
Cefuroxime	31.25% (5/16)	56.16% (41/73)	62.83% (71/113)	63.46% (33/52)	47.49% (104/219)	53.70% (254/473)
Cefuroxime (Oral)	31.25% (5/16)	49.32% (36/73)	59.29% (67/113)	61.54% (32/52)	40.64% (89/219)	48.41% (229/473)
Ceftriaxone (Non-meningitis)	12.50% (2/16)	15.28% (11/72)	13.51% (15/111)	20.00% (10/50)	16.13% (35/217)	15.67% (73/466)
Ceftriaxone (Meningitis)	–	0.00% (0/1)	50.00% (1/2)	0.00% (0/2)	50.00% (1/2)	28.57% (2/7)
Vancomycin	0.00% (0/16)	0.00% (0/74)	0.00% (0/113)	0.00% (0/52)	0.00% (0/219)	0.00% (0/474)
Erythromycin	93.75% (15/16)	97.30% (72/74)	94.69% (107/113)	88.00% (44/50)	94.04% (205/218)	94.06% (443/471)
Azithromycin	93.75% (15/16)	97.26% (71/73)	92.04% (104/113)	78.85% (41/52)	94.52% (207/219)	92.60% (438/473)
Clarithromycin	93.75% (15/16)	93.15% (68/73)	92.04% (104/113)	82.69% (43/52)	93.12% (203/218)	91.74% (433/472)
Tetracycline	87.50% (14/16)	91.78% (67/73)	88.50% (100/113)	94.23% (49/52)	88.58% (194/219)	89.64% (424/473)
Levofloxacin	0.00% (0/16)	2.70% (2/74)	1.77% (2/113)	3.85% (2/52)	2.74% (6/219)	2.53% (12/474)
Moxifloxacin	0.00% (0/16)	0.00% (0/74)	0.00% (0/113)	0.00% (0/52)	0.91% (2/219)	0.42% (2/474)
Trimethoprim-Sulfamethoxazole	68.75% (11/16)	65.75% (48/73)	65.49% (74/113)	51.92% (27/52)	52.29% (114/218)	58.05% (274/472)
Chloramphenicol	12.50% (2/16)	8.11% (6/74)	10.62% (12/113)	5.77% (3/52)	7.76% (17/219)	8.44% (40/474)
Clindamycin	93.75% (15/16)	97.26% (71/73)	89.38% (101/113)	67.31% (35/52)	88.53% (193/218)	87.92% (415/472)
MDR	93.75% (15/16)	97.26% (71/73)	92.92% (105/113)	90.00% (45/50)	94.95% (207/218)	94.06% (443/471)

**Table 5:** Antibiotic resistance of *S. pneumoniae* by disease category

Antibiotic	Invasive			Non-invasive		
	Number of resistant strains	Total number	Resistant rate (%)	Number of resistant strains	Total number	Resistant rate (%)
Penicillin (Meningitis)	4	7	57.14	0	0	–
Penicillin (Non-meningitis)	1	44	2.27	13	422	3.08
Penicillin (Oral)	15	51	29.41	202	422	47.87
Amoxicillin	6	51	11.76	73	422	17.30
Cefaclor	25	51	49.02	278	422	65.88
Cefuroxime	20	51	39.22	234	422	55.45
Cefuroxime (Oral)	19	51	37.25	210	422	49.76
Ceftriaxone (Non-meningitis)	6	44	13.64	67	422	15.88
Ceftriaxone (Meningitis)	2	7	28.57	0	0	–
Vancomycin	0	51	0.00	0	423	0.00
Erythromycin	48	51	94.12	395	420	94.05
Azithromycin	46	51	90.20	392	422	92.89
Clarithromycin	46	51	90.20	387	421	91.92
Tetracycline	49	51	96.08	375	422	88.86
Levofloxacin	2	51	3.92	10	423	2.36
Moxifloxacin	0	51	0.00	2	423	0.47
Trimethoprim-Sulfamethoxazole	25	51	49.02	249	421	59.14
Chloramphenicol	6	51	11.76	34	423	8.04
Clindamycin	42	51	82.35	373	421	88.60
MDR	47	51	92.16	396	420	94.29

### Antibiotic resistance profiles of *S. pneumoniae* by vaccine types

**Fig. 5:**
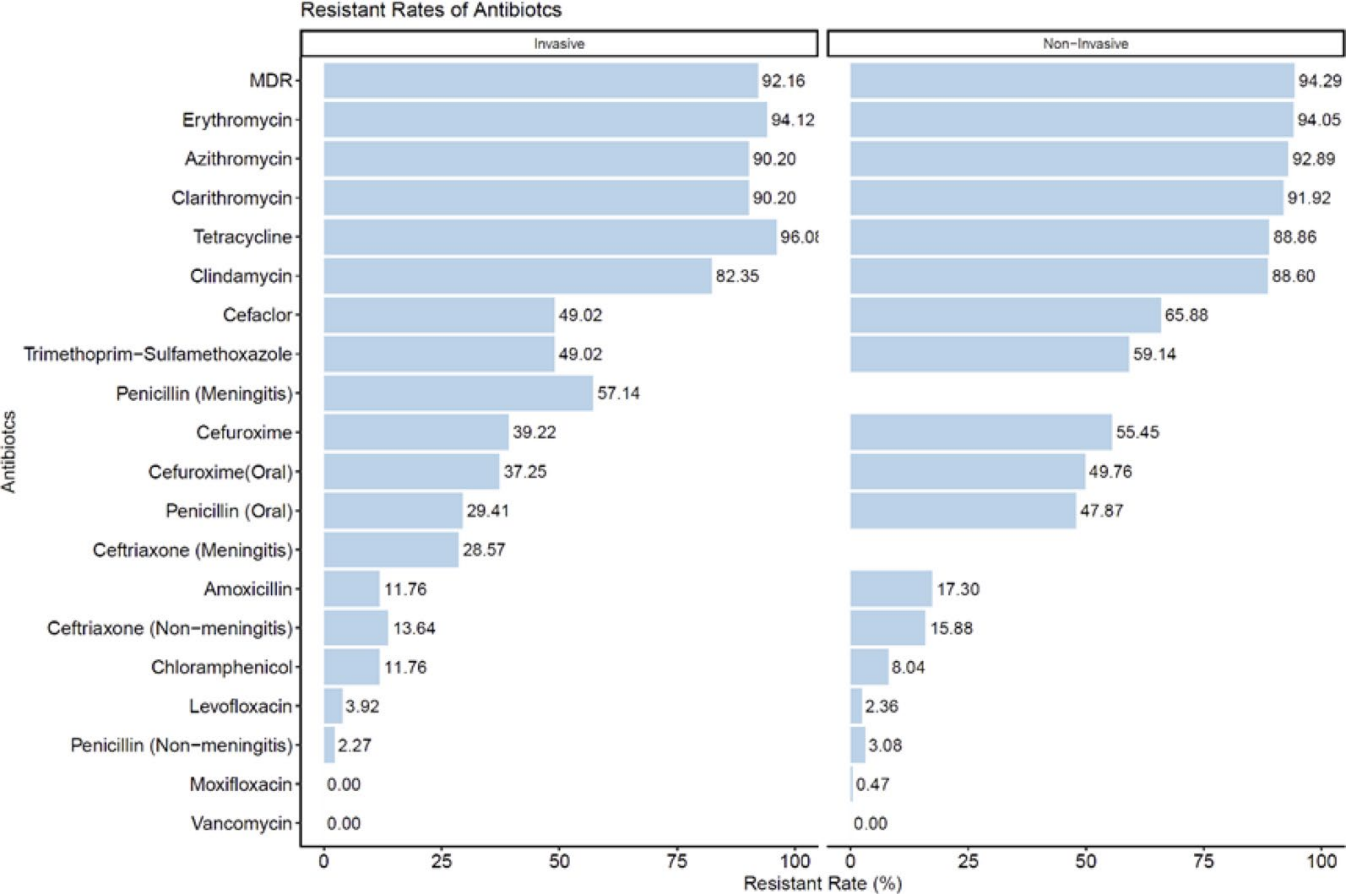
Antibiotic resistance of *S. pneumoniae* by disease category

Antibiotic resistance patterns differed between invasive and non-invasive *S. pneumoniae* isolates (Fig. 5). Overall, multidrug resistance (MDR) was highly prevalent in both groups, with slightly higher resistance rates observed in non-invasive isolates (94.29%) compared to invasive isolates (92.16%). Resistance to macrolides, including erythromycin, azithromycin, and clarithromycin, was consistently high, exceeding 90% in both invasive and non-invasive isolates. Tetracycline resistance was also substantial, reaching 96.08% in invasive isolates and 91.92% in non-invasive isolates. Clindamycin resistance followed a similar trend, with rates of 82.35% and 88.60% in invasive and non-invasive isolates, respectively.

For β-lactam antibiotics, cefaclor resistance was higher in non-invasive isolates (65.88%) than in invasive isolates (49.02%). Trimethoprim-sulfamethoxazole resistance was similar between the two groups (49.02% vs. 59.14%). When stratified by clinical syndrome and invasiveness, penicillin resistance was notably higher among isolates associated with meningitis—57.14% in invasive strains and 55.45% in non-invasive strains—compared to significantly lower resistance rates among non-meningitis-associated isolates (2.27% in invasive strains and 3.08% in non-invasive strains). Resistance to third-generation cephalosporins also varied: cefuroxime resistance was 39.22% in invasive isolates and 49.76% in non-invasive isolates, while ceftriaxone resistance among meningitis-related strains was 28.57% in invasive and 47.87% in non-invasive isolates. Fluoroquinolone resistance remained low, with levofloxacin resistance below 5% and moxifloxacin resistance nearly absent. Vancomycin resistance was not detected in any isolate.

**Fig. 6:**
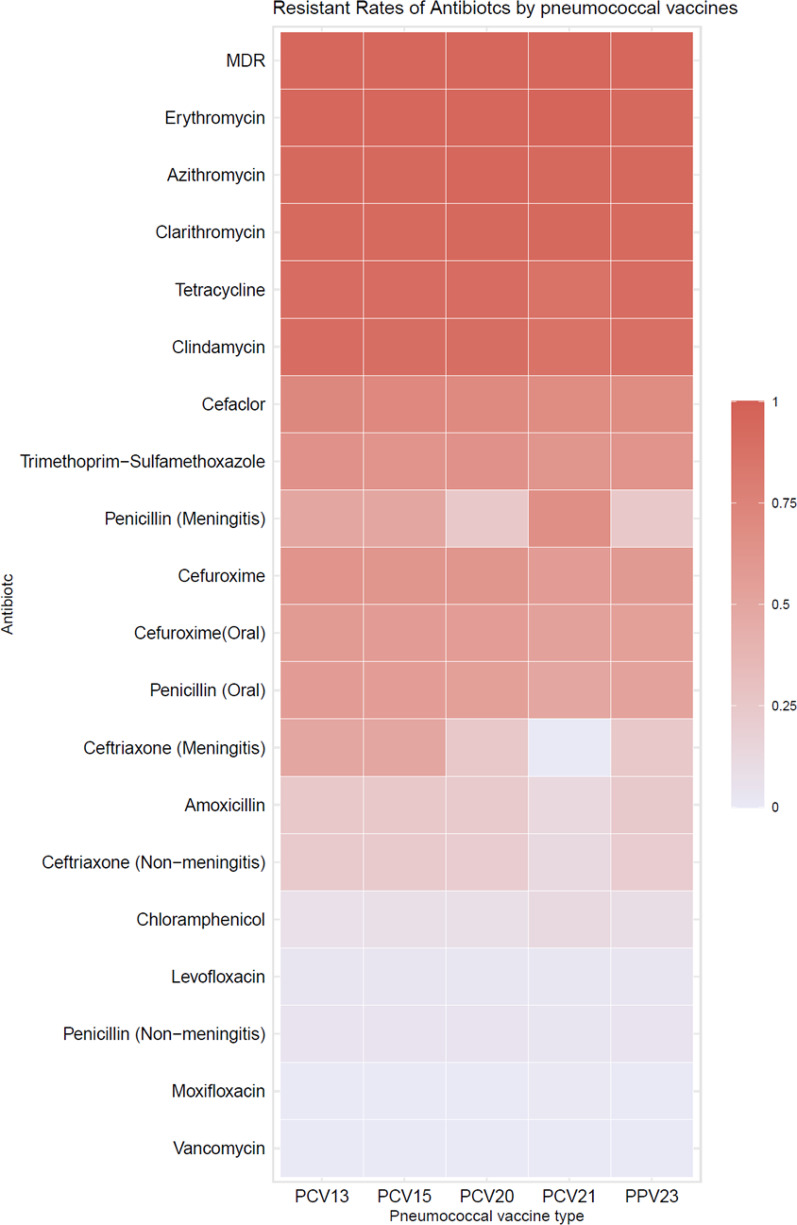
Antibiotic resistance of *S. pneumoniae* by vaccine types

When stratified by vaccine serotype coverage, resistance rates varied across pneumococcal vaccines (PCV13, PCV15, PCV20, PCV21, and PPV23) (Fig. 6). Serotypes covered by the higher-valency vaccines (PCV20 and PCV21) tended to exhibit higher resistance to macrolides, tetracyclines, and clindamycin, suggesting that resistant strains are more likely to be found within these serotypes. Penicillin resistance, particularly in meningitis-associated strains, remained moderate among serotypes covered by different vaccine formulations, while resistance to fluoroquinolones and vancomycin was consistently low across all vaccine serotype groups. Antibiotic resistance patterns among isolates stratified by vaccine coverage (PCV13, PCV15, PCV20, PCV21, PPV23) are presented in Table 6.

**Table 6:** Antibiotic resistance of *S. pneumoniae* by vaccine types

Antibiotic	PCV13 (%)	PCV15 (%)	PCV20 (%)	PCV21 (%)	PPV23 (%)
Penicillin (Meningitis)	50.00	50.00	25.00	66.67	25.00
Penicillin (Non-meningitis)	4.07	4.01	3.70	2.98	3.93
Penicillin (Oral)	56.57	55.81	54.27	50.29	51.78
Amoxicillin	25.25	24.92	23.17	13.45	23.62
Cefaclor	72.39	71.43	70.43	68.42	67.64
Cefuroxime	62.96	62.13	61.59	57.31	59.22
Cefuroxime (Oral)	57.91	57.14	56.40	52.63	54.37
Ceftriaxone (Non-meningitis)	23.05	22.74	20.99	11.90	21.31
Ceftriaxone (Meningitis)	50.00	50.00	25.00	0.00	25.00
Vancomycin	0.00	0.00	0.00	0.00	0.00
Erythromycin	94.61	94.68	94.82	96.47	94.50
Azithromycin	94.28	94.35	94.51	93.57	93.85
Clarithromycin	93.27	93.36	93.60	94.15	92.88
Tetracycline	90.91	90.70	91.46	87.13	91.26
Levofloxacin	2.68	2.65	2.43	1.75	2.58
Moxifloxacin	0.00	0.00	0.00	0.58	0.00
Trimethoprim-Sulfamethoxazole	63.64	63.12	64.33	61.99	62.78
Chloramphenicol	7.38	7.95	8.21	11.70	9.03
Clindamycin	91.25	90.37	90.24	86.55	89.32
MDR	95.29	95.35	95.43	95.29	94.82

## Discussion

In this multicenter study conducted in China, we evaluated the serotype distribution and antimicrobial susceptibility of *S. pneumoniae* isolates cultured from adult patients. A total of 474 pneumococcal isolates were obtained, with serotype 19F being the most prevalent (24.9%), followed by 19A (10.3%), 23F (9.5%) and 6A (5.9%), consistent with findings from previous studies conducted in China [19]. The predominance of serotype 19F across age groups, geographic regions, and specimen sources observed in this study highlights its continued circulation in the adult population in China. However, our results differ from those reported in other regions, as recent studies have highlighted notable geographic variation in pneumococcal serotype distribution. For instance, the predominant serotypes identified in the United States include 3, 22F, 19A, 35B, 9N, and 19F; in Korea, serotypes 3, 10A, 19A, and 23A are most frequently observed; whereas in Japan, 35B, 11A, and 3 are the most common [20–23]. One multi-center study from 2009 to 2015 when the PCV13 (including serotype 3) was not available in China reported that serotype 3 was the most predominant serotype (21.7%), followed by 19F, 19 A and 23F. However, we have not yet identify any studies with serotype 3 as a top serotype since the launch of PCV13 in China [23]. The observed overrepresentation of serotype 33F in invasive isolates suggests that this PCV20-nonPCV13 serotype may possess a higher invasive potential compared to its prevalence in non-invasive cases. This finding highlights the clinical relevance of emerging non-PCV13 serotypes and underscores the importance of ongoing surveillance to monitor potential shifts in pneumococcal serotype dynamics. Given that 33F is not covered by PCV13, its increasing presence among invasive cases may have implications for vaccine effectiveness and support the consideration of broader-valency vaccines in future immunization strategies.

With regard to vaccine coverage, PCV20 provided the highest overall coverage (69.4%) among all evaluated vaccines and consistently outperformed other formulations across all age groups and regions, except in the central region where PPV23 showed slightly higher coverage (75.0%). Notably, PCV20 also demonstrated the greatest coverage in both invasive and non-invasive isolates, suggesting its potential utility in broader adult immunization strategies. Similar to our findings, a study from Turkey reported that among adults aged 18–64 years, serotype coverage rates were 62.8% for PCV13, 65.1% for PCV15, 71.0% for PCV20, and 74.3% for PPV23. In adults aged ≥ 65 years, the corresponding coverage rates were 66.0%, 69.5%, 80.1%, and 78.7%, respectively [24].

Our study highlights the extensive antibiotic resistance among *S. pneumoniae* isolates from hospitalized adult patients in China, with particularly high rates of MDR and significant regional and strain-specific variations. These findings emphasize the urgent need for improved antimicrobial stewardship and vaccination strategies to mitigate the burden of antibiotic-resistant pneumococcal infections.

The observed MDR prevalence of 94.06% aligns with previous reports of high resistance rates in Asia [25]. Macrolide resistance exceeding 90% for erythromycin, azithromycin, and clarithromycin suggests widespread dissemination of resistance determinants, likely driven by the overuse of these antibiotics in outpatient settings [26]. Tetracycline (89.64%) and clindamycin (87.92%) resistance further reflect the impact of selective pressure from commonly prescribed antibiotics [27]. Conversely, low fluoroquinolone resistance and absence of vancomycin resistance provide critical insights into potential treatment options for severe pneumococcal infections [28].

The substantial variation in resistance rates across regions suggests distinct antibiotic prescribing practices and potential differences in circulating pneumococcal lineages. The East and West regions demonstrated higher resistance to β-lactam antibiotics, which may reflect differences in healthcare infrastructure, antibiotic use regulations, and local epidemiology. Notably, ceftriaxone resistance was lowest in the East and highest in the West, highlighting potential geographic disparities in treatment efficacy.

MDR was slightly higher in non-invasive isolates (94.29%) compared to invasive isolates (92.16%), consistent with prior findings [29]. Unlike invasive isolates, which primarily cause bloodstream and cerebrospinal infections, non-invasive strains colonize the nasopharynx and are frequently exposed to subtherapeutic antibiotic levels, promoting horizontal gene transfer of resistance determinants [30]. Additionally, non-invasive pneumococcal strains often carry mobile genetic elements, such as transposons and plasmids, facilitating the acquisition of resistance genes (Cohen et al., 2019). Resistance patterns differed by syndrome, with higher penicillin resistance in meningitis-associated strains, particularly in invasive isolates (57.14%). This could be due to the limited penetration of β-lactam antibiotics into the cerebrospinal fluid (CSF), leading to subtherapeutic drug concentrations that select for resistant strains. The relatively lower resistance to third-generation cephalosporins (ceftriaxone and cefuroxime) suggests their continued role in treatment, albeit with caution due to rising resistance trends.

Serotype-specific resistance patterns indicate that strains covered by higher-valency pneumococcal vaccines (PCV20) exhibited greater macrolide, tetracycline, and clindamycin resistance, suggesting that antibiotic-resistant pneumococcal serotypes are more prevalent within vaccine-covered groups. This finding underscores the need for continued surveillance to assess the impact of serotype replacement and vaccine effectiveness in reducing antibiotic-resistant pneumococcal infections.

## Electronic supplementary material

Below is the link to the electronic supplementary material.

Supplementary Material 1

## Data Availability

The datasets used and/or analyzed during the current study are available from the corresponding author on reasonable request.
