# Supplementary material for: Distribution of serotypes and antibiotic resistance profiles of Streptococcus pneumoniae in hospitalized adult patients: aretrospective multicenter surveillance in China (2018–2019)
Source: BMC Infect Dis. 2025 Aug 5;25:980. doi: 10.1186/s12879-025-11377-5 (PMC12323133; doi:10.1186/s12879-025-11377-5)
Supplement: Supplementary file 1 — Supplementary Material 1 [file 12879_2025_11377_MOESM1_ESM.docx]

Table S1. Characteristics of non-invasive and invasive *S. pneumoniae* isolates

| Characteristics | Non-invasive | |  | Invasive | |
| --- | --- | --- | --- | --- | --- |
|  | No. | % |  | No. | % |
| Total | 423 | 100.0 |  | 51 | 100.0 |
| Specimen source |  |  |  |  |  |
| Sputum | 334 | 79.0 |  | 0 | 0.0 |
| Bronchoalveolar lavage | 52 | 12.3 |  | 0 | 0.0 |
| Nose | 14 | 3.3 |  | 0 | 0.0 |
| Secretions | 5 | 1.2 |  | 0 | 0.0 |
| Brush biopsy | 4 | 0.9 |  | 0 | 0.0 |
| Sinus | 4 | 0.9 |  | 0 | 0.0 |
| Pus | 3 | 0.7 |  | 0 | 0.0 |
| Urine | 3 | 0.7 |  | 0 | 0.0 |
| Drainage | 2 | 0.5 |  | 0 | 0.0 |
| Ear | 1 | 0.2 |  | 0 | 0.0 |
| Throat swab | 1 | 0.2 |  | 0 | 0.0 |
| Blood | 0 | 0.0 |  | 41 | 80.4 |
| Cerebrospinal fluid | 0 | 0.0 |  | 7 | 13.7 |
| Eye (Sterile) | 0 | 0.0 |  | 2 | 3.9 |
| Pleural fluid | 0 | 0.0 |  | 1 | 2.0 |
| Region (city, province) |  |  |  |  |  |
| East | 65 | 15.4 |  | 9 | 17.6 |
| Dong'a County People's Hospital (Dong’a, Shandong) | 19 | 4.5 |  | 3 | 5.9 |
| Shandong Provincial Hospital (Jinan, Shandong) | 21 | 5.0 |  | 1 | 2.0 |
| Taian Central Hospital (Taian, Shandong) | 25 | 5.9 |  | 5 | 9.8 |
| South | 44 | 10.4 |  | 8 | 15.7 |
| Guangzhou Institute of Respiratory Health (Guangzhou, Guangdong) | 18 | 4.3 |  | 2 | 3.9 |
| Liuzhou People's Hospital (Liuzhou, Guangxi) | 24 | 5.7 |  | 6 | 11.8 |
| Liuzhou Maternal and Child Health Hospital (Liuzhou, Guangxi) | 1 | 0.2 |  | 0 | 0.0 |
| The First Affiliated Hospital of Sun Yat-sen University (Guangzhou, Guangdong) | 1 | 0.2 |  | 0 | 0.0 |
| West | 101 | 23.9 |  | 12 | 23.5 |
| Ningxia Medical University General Hospital (Yinchuan, Ningxia) | 27 | 6.4 |  | 1 | 2.0 |
| The Third Hospital of Mianyang (Mianyang, Sichuan) | 39 | 9.2 |  | 3 | 5.9 |
| West China Hospital of Sichuan University (Chengdu, Sichuan) | 16 | 3.8 |  | 6 | 11.8 |
| Xijing Hospital (Xian, Shannxi) | 19 | 4.5 |  | 2 | 3.9 |
| North | 199 | 47.0 |  | 20 | 39.2 |
| Beijing Tsinghua Chang Gung Memorial Hospital (Beijing) | 5 | 1.2 |  | 1 | 2.0 |
| Beijing Chaoyang Hospital (Beijing) | 72 | 17.0 |  | 7 | 13.7 |
| China-Japan Friendship Hospital (Beijing) | 37 | 8.7 |  | 3 | 5.9 |
| China Medical University Shengjing Hospital (Shenyang, Liaoning) | 15 | 3.5 |  | 4 | 7.8 |
| Jilin University Sino-Japanese Friendship Hospital (Changchun, Jilin) | 3 | 0.7 |  | 0 | 0.0 |
| Peking University People's Hospital (Beijing) | 67 | 15.8 |  | 5 | 9.8 |
| Central | 14 | 3.3 |  | 2 | 3.9 |
| Wuhan Fourth Hospital (Wuhan, Hubei) | 11 | 2.6 |  | 2 | 3.9 |
| Xiangya Hospital Central South University (Changsha, Hunan) | 1 | 0.2 |  | 0 | 0.0 |
| Hunan Provincial People's Hospital (Changsha, Hunan) | 2 | 0.5 |  | 0 | 0.0 |

Table S2. Distribution of vaccine-covered serotypes among invasive and non-invasive Isolates

| Serotype | Total  (n=474) | Specimen source | | χ^2^ | *P* value |
| --- | --- | --- | --- | --- | --- |
|  |  | Invasive  (n=51) | Non-invasive  (n=423) |  |  |
| 1 | 2 (0.4) | 1 (2.0) | 1 (0.2) | / | 0.204^a^ |
| 3 | 7 (1.5) | 1 (2.0) | 6 (1.4) | / | 0.552^a^ |
| 4 | 1 (0.2) | 1 (2.0) | 0 (0.0) | / | 0.108^a^ |
| 5 | 1 (0.2) | 0 (0.0) | 1 (0.2) | / | 1^a^ |
| 6A | 28 (5.9) | 6 (11.8) | 22 (5.2) | 2.446 | 0.118 |
| 6B | 10 (2.1) | 1 (2.0) | 9 (2.1) | 0.001 | 1 |
| 7F | 6 (1.3) | 1 (2.0) | 5 (1.2) | / | 0.497^a^ |
| 8 | 2 (0.4) | 0 (0.0) | 2 (0.5) | / | 1^a^ |
| 9N | 1 (0.2) | 0 (0.0) | 1 (0.2) | / | 1^a^ |
| 9V | 7 (1.5) | 1 (2.0) | 6 (1.4) | / | 0.552^a^ |
| 10A | 4 (0.8) | 0 (0.0) | 4 (0.9) | / | 1^a^ |
| 11A | 13 (2.7) | 1 (2.0) | 12 (2.8) | 0.001 | 1 |
| 12F | 1 (0.2) | 0 (0.0) | 1 (0.2) | / | 1^a^ |
| 14 | 20 (4.2) | 3(5.9) | 17 (4.0) | 0.066 | 0.797 |
| 15A | 25 (5.3) | 0 (0.0) | 25 (5.9) | 2.109 | 0.146 |
| 15B | 7 (1.5) | 1 (2.0) | 6 (1.4) | / | 0.552^a^ |
| 15C | 2 (0.4) | 0 (0.0) | 2 (0.5) | / | 1^a^ |
| 16F | 3 (0.6) | 0 (0.0) | 3 (0.7) | / | 1^a^ |
| 17F | 2 (0.4) | 1 (2.0) | 1 (0.2) | / | 0.204^a^ |
| 18C | 4 (0.8) | 0 (0.0) | 4 (0.9) | / | 1^a^ |
| 19A | 49 (10.3) | 6 (11.8) | 43 (10.2) | 0.126 | 0.723 |
| 19F | 118 (24.9) | 14 (27.5) | 104 (24.6) | 0.200 | 0.655 |
| 20 | 6 (1.3) | 0 (0.0) | 6 (1.4) | / | 1^a^ |
| 22F | 1 (0.2) | 0 (0.0) | 1 (0.2) | / | 1^a^ |
| 23A | 9 (1.9) | 1 (2.0) | 8 (1.9) | / | 1^a^ |
| 23B | 6 (1.3) | 1 (2.0) | 5 (1.2) | / | 0.497^a^ |
| 23F | 45 (9.5) | 2 (3.9) | 43 (10.2) | 1.402 | 0.236 |
| 31 | 1 (0.2) | 0 (0.0) | 1 (0.2) | / | 1^a^ |
| 33F | 3 (0.6) | 2 (3.9) | 1 (0.2) | / | 0.032^a^ |
| 35B | 2 (0.4) | 0 (0.0) | 2 (0.5) | / | 1^a^ |

^a^ Fisher's exact test.
